# Refining the In-Parameter-Order Strategy for Constructing Covering Arrays

**DOI:** 10.6028/jres.113.022

**Published:** 2008-10-01

**Authors:** Michael Forbes, Jim Lawrence, Yu Lei, Raghu N. Kacker, D. Richard Kuhn

**Affiliations:** National Institute of Standards and Technology, Gaithersburg, MD 20899; Massachusetts Institute of Technology, Cambridge, MA 02139; National Institute of Standards and Technology, Gaithersburg, MD 20899; George Mason University, Fairfax, VA 22030; University of Texas at Arlington, Arlington, TX 76019; National Institute of Standards and Technology, Gaithersburg, MD 20899

**Keywords:** blackbox testing, covering arrays, pairwise and higher strength testing

## Abstract

Covering arrays are structures for well-representing extremely large input spaces and are used to efficiently implement blackbox testing for software and hardware. This paper proposes refinements over the In-Parameter-Order strategy (for arbitrary *t*). When constructing homogeneous-alphabet covering arrays, these refinements reduce runtime in nearly all cases by a factor of more than 5 and in some cases by factors as large as 280. This trend is increasing with the number of columns in the covering array. Moreover, the resulting covering arrays are about 5 % smaller. Consequently, this new algorithm has constructed many covering arrays that are the smallest in the literature. A heuristic variant of the algorithm sometimes produces comparably sized covering arrays while running significantly faster.

## 1. Introduction

For the purposes of this paper, a *v-valued n* × *k covering array of strength t* with integer parameters *n*, *k*, *t*, and *v*, where *v*, *t* ≥ 2, *k* ≥ *t*, and *n* > 0 is a matrix *C* of size *n* × *k* with entries from {0, 1, …, *v* − 1} which has the property that each submatrix of size *n* × *t* has among its rows all of the *v^t^* possible tuples (*x*_1_, …, *x_t_*) of integers where 0 ≤ *x_i_* < *v* for each index *i* ∈ {1, …, *t*}. For the rest of this paper the four parameters will be implicit and we will just refer to such arrays as *covering arrays*. As the value of *v* is constant over the columns of the array, this is a *homogeneous alphabet* covering array. This concept can be generalized to *heterogeneous alphabets* so that each column, *j*, has a different *v_j_*, but this paper will not discuss such cases as while all ideas presented apply, the empirical results have not been well explored.

A set Λ of *t* indices *j*_1_, …, *j_t_*, where 1 ≤ *j*_1_ < ⋯ < *j_t_* ≤ *k*, together with a function *ν* on Λ such that *ν* (*j*) ∈ {0, …, *v* − 1} for each *j* ∈ Λ will be called a *t*-tuple, with Λ referred to as the column tuple and *ν* the value tuple. A row (*x*_1_,. …, *x_k_*) is said to cover a *t*-tuple (Λ, *ν*) provided that *x_j_* = *ν* (*j*) for each *j* ∈ Λ. Thus *C* is a covering array if and only if each *t*-tuple is covered by at least one row in *C*.

Software testing is often done with test inputs sampled from a large input space. Taking each row in the covering array as a *test* from a sample space of size *v^k^* allows covering arrays to identify which test inputs should be used to check software validity. This is desirable because covering arrays well-represent the full sample space by covering all *t*-tuples. Theoretical results [3] tell us that covering arrays need not require more than approximately *tv^t^* log(*vk*) tests, which is drastically less than the full-testing that covering arrays approximate. Thus, covering arrays are used as an efficient way for picking tests for software [5,7,9]. For a survey of covering arrays in the binary (*v* = 2) case, see [6].

Several algorithms for constructing covering arrays suitable for software testing have been developed. Some use the “In-Parameter-Order,” or IPO, strategy [7,9]. Here, some refinements of this strategy are proposed and studied. There are two competing goals for algorithms that construct covering arrays: to minimize the time required to produce the array, and to minimize the number of rows, *n*, in the array. In this paper we present changes to IPO which empirically reduce both the execution time and the resulting covering array size.

The original IPO strategy was implemented for 2-way coverage, that is, for *t* = 2. However, the principle behind the algorithm of treating the columns (parameters) one by one applies for all *t*, and the IPO strategy is being used as a starting point for the efficient production of covering arrays for values of *t* up to 6. This more general endeavor is designated IPOG (“In-Parameter-Order-Generalized”), which is a centerpiece of the *Automated Combinatorial Testing for Software* project at the National Institute of Standards and Technology (NIST). See [8,10] for current information on IPOG. The new strategy incorporates some modifications to IPOG which are intended as an aid in constructing covering arrays for this project. Some results of a preliminary evaluation of these ideas are presented. The tables of covering arrays [11] are products of the use of these ideas.

## 2. The IPO Framework

We briefly describe the operation of IPO. Unlike many other algorithms that build covering arrays one row at a time, the IPO strategy builds covering arrays one column at a time. Specifically, it uses the idea that covering arrays of *k* − 1 parameters can be used to efficiently build a covering array of *k* parameters. Applying this induction with the trivial base case *k* = *t* allows for generating any covering array desired.

To construct the covering array, first make a matrix for the first *t* parameters which contains each of the possible *v^t^* distinct rows having entries from {0, …, *v* − 1}. This matrix will be of size *v^t^* × *t*. Then, for each additional parameter, perform the following two steps.

- *Horizontal growth:* Add an additional column (corresponding to the new parameter) and fill in its values.
- *Vertical growth:* For each column tuple, if some value tuple fails to appear, add a new row to cover this *t*-tuple.

There are many ways to implement the procedures for horizontal and vertical growth. Horizontal growth procedures must address which values are assigned to each entry in the new column. The greedy idea of choosing values that maximize the number of covered *t*-tuples has been shown to produce fairly small covering arrays in an efficient manner, but there is still the question of which order to fill in the entries in the new column. Two options are presented in [7], one uses the row order and another tries all possible orders. This paper will explore a third option which greedily picks the row order.

Vertical growth algorithms must decide how to add rows onto a covering array in a way that covers the *t*-tuples not covered by horizontal growth. One method, as described in [7], is to add rows that are specified with only as much detail as desired, such as to cover a specific *t*-tuple, while leaving the rest of the row filled with “don’t care” values. That is, the symbol *θ* may appear as an entry in the matrix in addition to the integers 0, …, *v* − 1, indicating that the value of that entry from {0, …, *v* − 1} has not yet been determined. Using *θ* allows the algorithm to defer this determination until more information is present. The *θ* values are replaced with integers either during the same stage of vertical growth or perhaps during another stage of vertical growth after more parameters have been added to the array.

In case *t* = 2, there is a natural method of implementing the vertical growth procedure, as described in [7]. For each value *x* from {0, …, *v* − 1} such that there exists a pair of columns (one necessarily the last) not containing all of the *v* value tuples having *x* in the last column, add a row to the matrix which has *x* in the last column and each earlier column has a value such that the 2-tuple composed of this value with *x* has not been covered, or if there is no such value, has *θ*. Denoting *f* (*x*) the maximum number of such other values in any other column, we see that at most Σ*_x_ f* (*x*) ≤ *v*^2^ new rows will be added at this stage. Furthermore, it is clear that in any vertical growth algorithm extending the already present rows, at least Σ*_x_ f* (*x*) rows are required. In this sense the procedure is optimal. The procedure, but not the sense of optimality, has been extended in the IPOG algorithm for arbitrary *t*: by induction of coverage over the columns, we only need to examine *t*-tuples with *k* ∈ Λ, so for each value of *ν* we determine whether that *t*-tuple (Λ, *ν*) is covered in the array. There would be $v^{t}\left(\begin{array}{l} k-1\\ t-1 \end{array}\right)$ such *t*-tuples. Any uncovered *t*-tuple would be added to the array by placing it directly in the array by replacing entries with a *θ* or by appending a row filled with *θ* to the array and then inserting the *t*-tuple. This paper primarily focuses on changes to the horizontal growth algorithm, but some minor changes to the vertical growth algorithm are also discussed.

## 3. The Main Algorithm

### 3.1 An Explanation of the Algorithm

The original IPO algorithm is composed of the horizontal growth stage and the vertical growth stage, and ideally we know good algorithms for both stages. However, horizontal growth constitutes most of the runtime and intuitively can be seen to be the critical factor in determining how small the resulting covering array is, as it determines how many *t*-tuples, and thus rows, vertical growth must place. When *t* = 2, as we saw above, Σ*_x_ f* (*x*) was the number of rows added by vertical growth, but it was determined by the horizontal, not vertical, growth stage. Thus, this paper seeks to explore the more important of the two stages in IPO by showing how broadening the search space of horizontal growth can increase the optimality of the results and decrease runtime.

The horizontal growth stage takes a covering array of *k* − 1 columns and extends it to an array of *k* columns by adding one column to the old array, thereby “extending” each row with some value. Any remaining uncovered *t*-tuples will be covered in the vertical growth stage. The choice of which rows will be extended with which values is the critical step in how any algorithm following this framework operates. The original IPO algorithm examines the rows in order and greedily selects the value to extend each row with. This paper outlines an algorithm that allows for greedy selection over both the row and value with which we extend the array. The metric for the greedy selection is unchanged: we want to pick an extension of the array that covers as many previously uncovered *t*-tuples as possible.

This generalization offers the possibility of producing smaller covering arrays by virtue of the larger search space for the greedy choice. At first glance it seems unlikely that this approach can achieve a practical runtime. A naive implementation that simply broadens the search space without major algorithmic alterations would incur a large performance cost because each time an extension is performed, all row/value pairs would be checked for the additional coverage they offer. $\left(\begin{array}{l} k-1\\ t-1 \end{array}\right)$ *t*-tuples may have their coverage affected by the extension and under a naive approach, all must be checked. This implementation would thus have a run-time of $\Theta \left(vr^{2}\left(\begin{array}{l} k-1\\ t-1 \end{array}\right)\right)$ as opposed to the better runtime of the original strategy, which is $\Theta \left(vr\left(\begin{array}{l} k-1\\ t-1 \end{array}\right)\right)$.

Fortunately, the naive implementation performs more work than necessary and can be improved. In the expanded search space, any algorithm must examine row/value pairs for their possible additional coverage multiple times, but a naive approach simply performs the calculation again. By using dynamic programming to store and update this information appropriately this larger search space can be explored much faster.

Consider a non-extended row. Extending it with some value will cover $\left(\begin{array}{l} k-1\\ t-1 \end{array}\right)$ *t*-tuples, some of which may have already been covered and others which would be newly covered. We can express this relationship with

$$t_{c}+t_{n}=\left(\begin{array}{l} k-1\\ t-1 \end{array}\right)$$ (1)

with *t_c_* denoting the “covered” *t*-tuples that have previously been covered by already extended rows and *t_n_* denoting the number of new *t*-tuples the row/value pair would cover if we choose that extension. As we want to maximize additional coverage, *t_n_* is the metric used to gauge which row/value pairs to extend the array with. The value of *t_n_* will not increase as we extend more rows in the array. The naive implementation directly maintains *t_n_*. However, if we use (1) with dynamic programming and maintain *t_c_* directly (and thus *t_n_* indirectly) we get the same greedy metric but, as will be shown, in a more efficient manner.

To maintain *t_c_* we have two arrays, *T_c_* [*r*, *v*] and *Cov*[Λ, *ν*]. *T_c_* is indexed by a row and a value and stores *t_c_* for this row/value pair. This takes Θ(*rv*) space. *Cov*[Λ, *ν*] is a boolean array indexed by the column tuple Λ and value tuple *ν* and entries indicate whether the *t*-tuple (Λ, *ν*) is covered. The IPO framework guarantees that the first *k* − 1 columns form a covering array, so we only need to consider the *t*-tuples that have the last column in their column tuple, Λ; therefore the array *Cov* takes $\Theta \left(v^{t}\left(\begin{array}{l} k-1\\ t-1 \end{array}\right)\right)$ space. Initially, *T_c_* is filled with zeros and *Cov* is filled with false’s. When the greedy selection occurs and a row is extended with a certain value, both of the arrays must be updated.

We take a brief moment to discuss how the arrays are indexed. *T_c_* is straightforward, but *Cov* is more complex as Λ and *ν* need an efficient scheme to be represented as numbers for *Cov* to be a conventional array. To do this, we note that combinations can be lexicographically ordered and the position of a combination Λ can be used as its numerical hash for accessing the array; see Knuth’s Theorem L [4]. As we have a homogeneous alphabet covering array we treat *ν* as a number with base *v*.

Consider when we just extended the row *i* ∈ {1, …, *n*} with the value *a* ∈ {0, …, *v* − 1}, which we denote as having performed the row extension (*i*, *a*). We then need to update the coverage count *T_c_*[*j*, *b*] for all other extensions (*j*, *b*), as well as which *t*-tuples have been covered as reflected by the *Cov* array.

*T_c_*[*j*, *b*] already has the “covered” *t*-tuple count for (*j*, *b*) prior to extending the array with the pair (*i*, *a*). We need to count among the $\left(\begin{array}{l} k-1\\ t-1 \end{array}\right)$ *t*-tuples the extension (*j*, *b*) covers which ones are “newly” covered by (*i*, *a*) and thus no longer contribute to the *t_n_* value for (*j*, *b*). A naive way to update *T* would be to check all $\left(\begin{array}{l} k-1\\ t-1 \end{array}\right)$ *t*-tuples. However, this offers no time savings over the naive implementation discussed earlier.

To achieve time savings, first notice that when we extended the array with (*i*, *a*), the only extensions (*j*, *b*) that can have their *t_c_* value change are those for which *b* = *a*. This is again from the inductive fact that the first *k* − 1 columns form a covering array so we are only considering *t*-tuples that have a Λ that includes the last column. So if *b* ≠ *a*, then the extension (*j*, *b*) cannot have any of its $\left(\begin{array}{l} k-1\\ t-1 \end{array}\right)$ *t*-tuples covered by the extension (*i*, *a*), so we need not update *T_c_*[*j*, *b*]. Thus, we only need to consider row/value pairs (*j*, *a*) when updating *T_c_*.

Second, instead of examining all $\left(\begin{array}{l} k-1\\ t-1 \end{array}\right)$ *t*-tuples, we can restrict ourselves to a smaller set. If a *t*-tuple (Λ, *ν*) was freshly covered by (*i*, *a*) and would also be covered by the possible row extension (*j*, *a*) then this means that for each *l* ∈ Λ, *ν* (*l*) is the entry in both positions (*i*, *l*) and (*j*, *l*) in the array. Therefore, the freshly covered *t*-tuples in row *j* are the *t*-tuples with a Λ that is a subset of the columns where row *i* and row *j* have identical entries.

This observation gives us the procedure we desire. Begin by examining the columns where the newly-extended row *i* and a row *j* have identical entries. Any freshly covered *t*-tuple in row *j* must have its column tuple entirely within these “shared columns.” If there are *s* shared columns, then we only need explore $\left(\begin{array}{c} s\\ t-1 \end{array}\right)$ values for Λ, as the last column must be in Λ. Notice that *ν* is completely specified by Λ and the two rows we are comparing. For each *t*-tuple “shared” between the two rows, we check *Cov*[Λ, *ν*] to see whether the *t*-tuple was covered previously. If so, this *t*-tuple doesn’t affect *T_c_*[*j*, *a*]. Otherwise, *T_c_*[*j*, *a*] is increased by one. These steps keep *T_c_* updated.

After updating *T_c_* for each non-extended row/value pair, *Cov* is updated by marking all $\left(\begin{array}{l} k-1\\ t-1 \end{array}\right)$ *t*-tuples covered by the extension (*i*, *a*) as “covered” if they were not so already.

With the update step for *T_c_* and *Cov* explained, the whole algorithm can be discussed. First, all non-extended rows are searched, calculating the *t_n_* values for each row/value pair from the *T_c_* array. A row/value pair is chosen greedily, with ties broken randomly, and that extension is performed. The update step then occurs and the process is repeated until either there are no rows to extend or no additional coverage would result from further extensions.

There are several nuances that still need to be discussed. Of principle concern is the search for the maximum *t_n_* value. Searching through all non-extended rows for the maximum *t_n_* value is wasteful. Recall that extending one row with a value *a* can only affect other row/value pairs that would also extend a row with the value *a*. Thus, the *t_n_* values only change on roughly $\frac{1}{v}$ of the iterations. Further, the *t_n_* values do not increase, so an intelligent data structure, such as a priority queue, could be used to exploit such properties in a highly efficient manner. However the large number of row/value pairs and the fact that we only extend with one pair for each row makes it seem wasteful in both time and space to maintain this data structure. Instead, a list of row/value pairs with the maximum *t_n_* value is maintained. Since the *t_n_* values do not increase the list can never get bigger until it completely runs out. Thus, instead of searching all row/value pairs, we simply pick a random candidate off the list and then prune the list of those row/value pairs which had their *t_n_* value decrease due to result of the update to *T_c_*. When the list runs out, we search all of the row/value pairs to find those that attain the maximum *t_n_* value. Experimental evidence suggests that for values of *v* of at least 10, the list runs out infrequently. For small values of *v* the lists’ size of *O* (*rv*) seems small enough to be manageable. Thus a better data structure seems unwarranted overall.

Another nuance is the presence of the “don’t care” values. These entries are unspecified so far because no additional coverage for the subarray of *k* − 1 parameters could be gained by specifying its value. They clearly have potential for additional coverage for the *k* parameters. To incorporate this potential into the *t_n_* values, one possible method would be to treat the don’t-care values in a way that assumes they maximize their potential. This seems difficult to implement in an efficient manner, and in particular, does not seem to fit in well with (1), the equation driving this entire approach. Instead, the choice was made that the potential of don’t-care values will be ignored during horizontal growth and if possible, don’t-care values will be replaced during vertical growth. To achieve this, we restate the relation as $t_{n}+t_{c}=\left(\begin{array}{c} g\\ t-1 \end{array}\right)$, where *g* is the number of “good”, or specified, columns in the row (excluding the last column).

The vertical growth algorithm is virtually identical to the original idea in IPOG. However, since some rows may be non-extended in horizontal growth some slight modifications have been made. When a *t*-tuple needs to be covered in vertical growth, all rows are searched for a suitable position and the first match is taken. Don’t-care values are filled in accordingly. To save time, the search is started in the first row where there are don’t-care values because some part of the row must be unspecified for a *t*-tuple to be placed there.

### 3.2 Pseudocode for the Algorithm

Algorithm 1 presents pseudocode for horizontal growth. For simplicity, the pseudocode does not implement the list of candidate row/value pairs and does not address the don’t-care values.

**Algorithm 1:** Horizontal Growth

*T_c_*[*i*, *a*] ← 0, ∀*i*, *a*
*Cov*[Λ, *ν*] ← *false*, ∀Λ, *ν*
**while** some row is non-extended **do**
Find non-extended row *i* and value *a* so that $t_{n}=\left(\begin{array}{l} k-1\\ t-1 \end{array}\right)-T_{c}\left[i,a\right]$ is maximum
**if** *t_n_* = 0 **then**
stop horizontal growth
**end if**
Extend row *i* with value *a*
**for all** non-extended rows *j* **do**
*S* ← set of columns where row *i* and *j* have identical entries
**for all** column tuples Λ ⊂ *S* **do**
*ν* ← the value tuple in row *i* and column tuple Λ
**if** *Cov*[Λ, *ν*] = *false* **then**
*T_c_*[*j*, *a*] ← *T_c_*[*j*, *a*] + 1
**end if**
**end for**
**end for**
**for all** column tuples Λ **do**
*ν* ← the value tuple in row *r* and column tuple Λ
**if** *Cov*[Λ, *ν*] = *false* **then**
*Cov*[Λ, *ν*] ← *true*
**end if**
**end for**
**end while**

Algorithm 2 presents pseudocode for vertical growth. With this pseudocode we can discuss further implementation details. Examine *T*_Λ_. One could simply use the array *Cov* from horizontal growth to determine which *t*-tuples are uncovered. However this approach only works for *t* = 2, as for general *t* when a *t*-tuple is placed in the array it could also create new coverage that was unintended. It is important to capture the unintended coverage, so we cannot just use *Cov*. Instead we calculate *T*_Λ_ for each Λ and doing so fully captures all coverage. A naive implementation to calculating *T*_Λ_ would require searching all rows and calculating the value of *ν* specified by that row and the given value of Λ, and removing the *t*-tuple (*ν*, Λ) from a list of *t*-tuples to cover. However, we get this information faster by traversing the column tuples Λ in a structured way. Using recursion, we can traverse the column tuples in a lexicographic order which infrequently changes many of the columns in Λ. Thus, for a given row, the numerical hash of *ν* that results also changes in a structured way as the entries in the value tuple also infrequently change. This clearly can be exploited to achieve time savings but the details will not be presented.

**Algorithm 2:** Vertical Growth

**for all** column tuples Λ : *k* ∈ Λ **do**
*T*_Λ_ ← list of uncovered *t*-tuples with this Λ
**for all** *ν* : (Λ, *ν*) ∈ *T*_Λ_ **do**
**for all** rows *i* with a don’t-care entry with a column in Λ **do**
**if** we can place (Λ, *ν*) **then**
place (Λ, *ν*)
**end if**
**end for**
**if** (Λ, *ν*) not placed yet **then**
add a new row with (Λ, *ν*) as the only entries
**end if**
**end for**
**end for**

### 3.3 Analysis

The space complexity of this algorithm was discussed above, with the requirements mainly driven by the two arrays *T_c_* and *Cov*, requiring Θ(*rv*) and $\Theta \left(v^{t}\left(\begin{array}{l} k-1\\ t-1 \end{array}\right)\right)$ space respectively. We now turn to the time analysis.

The performance of the algorithm in practice has proven to be very competitive as will be discussed in Sec. 5. Theoretically the properties of this algorithm are less clear. The complexity of the algorithm suggests no optimality guarantee is possible and unfortunately even a rigorous time bound is elusive. While the algorithm is not randomized (except for minor portions), the notion of “average” is needed here since what the worst-case input would be is a property of covering arrays that is unknown. Thus, we explore the runtime using some heuristic arguments.

Let’s first look at one stage of the horizontal growth algorithm. For each non-extended row, we first must search in *O* (*rv*) time for the extension (*i*, *a*) to perform. The candidate list has not been thoroughly explored to give a better guarantee. Then, for each non-extended row, *j*, we must explore the $\left(\begin{array}{c} s\\ t-1 \end{array}\right)$ *t*-tuples needed to update *T_c_*[*j*, *a*]. Calculating the *ν* required to index *Cov* seems like it might take Θ(*t*) time, but the lexicographic ordering mentioned earlier allows this to be done in amortized Θ(1) time so exploring the *t*-tuples takes $\Theta \left(\left(\begin{array}{c} s\\ t-1 \end{array}\right)\right)$. We then iterate through the $\left(\begin{array}{l} k-1\\ t-1 \end{array}\right)$ *t*-tuples (*i*, *a*) covers in order to update *Cov*. This gives the time guarantee of $O\left(\sum_{i=1}^{r}\left(rv+\left({}_{t-1}^{k-1}\right)+\sum_{j=i+1}^{r}\left({}_{t-1}^{s}\right)\right)\right)=O\left(r^{2}v+r\left({}_{t-1}^{k-1}\right)+r^{2}\bar{S}\right)=O\left(r\left({}_{t-1}^{k-1}\right)+r^{2}\bar{S}\right)$ with $\bar{S}$ the average $\left(\begin{array}{c} s\\ t-1 \end{array}\right)$ value. A rough guess at the value of $\bar{S}$ would take the fact that *s* ≤ *k* − 1 to have $\bar{S}\leq \left(\begin{array}{l} k-1\\ t-1 \end{array}\right)$. This would give $O\left(r\left(\begin{array}{l} k-1\\ t-1 \end{array}\right)+r^{2}\left(\begin{array}{l} k-1\\ t-1 \end{array}\right)\right)$. But this is largely unsatisfactory as the original IPO framework takes $O\left(rv\left({}_{t-1}^{k-1}\right)\right)$ time. This shows that the algorithm suffers from a large drawback because of this *r*^2^ term, which begs the question of whether a better bound for $\bar{S}$ can be found.

A better analysis returns to the key step of the algorithm where we update *T_c_* for each non-extended row, *j*. The number of *t*-tuples that are examined in this step is $\left(\begin{array}{c} s\\ t-1 \end{array}\right)$, but this is clearly dependent on the row that was last extended, *i*, and the row being updated, *j*, as *s* is the number of columns that have identical entries. With this step dominating much of the runtime of the algorithm it is critical to do a thorough analysis, and yet because of the extreme uncertainty in this property of covering arrays, it seems unlikely any rigorous argument can be made other than $\left(\begin{array}{c} s\\ t-1 \end{array}\right)\leq \left(\begin{array}{l} k-2\\ t-1 \end{array}\right)$. *k* − 2 is used because by the inductive step we know the first *k −* 1 columns are a covering array made by this algorithm, hence no two rows are exactly the same. However, small sizes of the resulting covering arrays suggests that the rows must be highly dissimilar so we suspect on average that $\left(\begin{array}{c} s\\ t-1 \end{array}\right)\ll \left(\begin{array}{l} k-2\\ t-1 \end{array}\right)$.

To deal with this, it seems reasonable to consider what would be the case if the first *k* − 1 columns were not a covering array, but instead were a random array. With this, we can take $E\left[\left(\begin{array}{c} s\\ t-1 \end{array}\right)\right]$ as an approximation of the number of *t*-tuples that will be explored in this part of the algorithm. While it should be clear that the algorithm will examine many fewer *t*-tuples than this because we are dealing with covering arrays, no rigorous argument for this fact is made. Using indicator random variables we get $E\left[\left(\begin{array}{c} s\\ t-1 \end{array}\right)=\frac{1}{v^{t-1}}\left(\begin{array}{l} k-1\\ t-1 \end{array}\right)\right]$, which is much less than the worst case bound of $\left(\begin{array}{l} k-2\\ t-1 \end{array}\right)$ in most cases. Using $\bar{S}\approx \frac{1}{v^{t-1}}\left(\begin{array}{l} k-1\\ t-1 \end{array}\right)$ we get the heuristic bound of $O\left(r\left(\begin{array}{l} k-1\\ t-1 \end{array}\right)+\frac{r^{2}}{v^{t-1}}\left(\begin{array}{l} k-1\\ t-1 \end{array}\right)\right)=O\left(\frac{r^{2}}{v^{t-1}}\left(\begin{array}{l} k-1\\ t-1 \end{array}\right)\right)$

For one stage of the vertical growth algorithm there is the clear worst case bound of $O\left(rv^{t}\left(\begin{array}{l} k-1\\ t-1 \end{array}\right)\right)$ which relies on the fact that we are only checking values of Λ that contain the last column. Realistically, the *v^t^* factor is an extremely weak upper bound as most *t*-tuples for any specific column tuple will be covered by the horizontal growth stage and thus no searching throughout the rows is performed. However, this argument doesn’t seem to lend itself to a better analysis.

To take a covering array of *k* − 1 parameters to *k* parameters we must combine both stages of the algorithm for a combined time bound of $O\left(\frac{r^{2}}{v^{t-1}}\left(\begin{array}{l} k-1\\ t-1 \end{array}\right)+rv^{t}\left(\begin{array}{l} k-1\\ t-1 \end{array}\right)\right)$. To get a total time bound for the algorithm we must generate the covering array from *t* columns up to the final *k*. Taking *r* as the final number of rows involved in the algorithm, this gives $O\left(\frac{r^{2}}{v^{t-1}}\left(\begin{array}{l} k\\ t \end{array}\right)+rv^{t}\left(\begin{array}{l} k\\ t \end{array}\right)\right)$.

We can take the heuristic argument one step further with the assumption, as suggested by experimental evidence in Fig. 3 and Fig. 6, that the resulting covering array size meets a logarithmic bound, in particular the bound $r\leq v^{t}\log\left(v^{t}\left(\begin{array}{l} k\\ t \end{array}\right)\right)$. The approximate time bound becomes $O\left(v^{t+1}\log^{2}\left(v^{t}\left(\begin{array}{l} k\\ t \end{array}\right)\right)\left(\begin{array}{l} k\\ t \end{array}\right)+v^{2t}\log\left(v^{t}\left(\begin{array}{l} k\\ t \end{array}\right)\right)\left(\begin{array}{l} k\\ t \end{array}\right)\right)$. While this bound does not itself suggest the algorithm is fast, experimental evidence to be presented in Sec. 5.2 does.

## 4. Heuristics

### 4.1 Modifying Horizontal Growth

One concern with computational approaches to covering array construction is their time-intensive nature. In this section we give a modification of the presented horizontal growth algorithm that aims to heavily reduce the time required while still producing decently sized covering arrays for smaller values of *v*, such as those less than ten.

To achieve this claim, we look at the step where *T_c_*[*j*, *a*] is updated and recall how it is this step that dominates much of the time in the algorithm. We had to examine $\left(\begin{array}{c} s\\ t-1 \end{array}\right)$ *t*-tuples to see if they were already covered, and if not, we performed the operation of setting *T_c_*[*j*, *a*] ← *T_c_*[*j*, *a*] + 1. It is easy to see then that *T_c_*[*j*, *a*] is incremented overall by no more than $\left(\begin{array}{c} s\\ t-1 \end{array}\right)$ and does not decrease. By using this information in an intelligent way we can avoid searching through any *t*-tuples at all.

The idea is to take *T_c_*[*j*, *a*] ← *T_c_*[*j*, *a*] + *f* (*n*, *s*, *t* − 1) with *n* as the number of already extended rows and *f* (*n*, *s*, *t* − 1) as a function that guesses how much *T_c_*[*j*, *a*] should increase without performing any searching at all. A prime candidate for *f* (*n*, *s*, *t* − 1) would be returning to the idea of a random array and using $E_{n}\left[\left(\begin{array}{c} s\\ t-1 \end{array}\right)\right]$, however this did not yield competitive covering array sizes. For reasons as yet unexplained, simply taking $f(n,s,t-1)=\left(\begin{array}{c} s\\ t-1 \end{array}\right)$, that is assuming that all *t*-tuples shared between row *i* and *j* were previously uncovered, yields competitive covering array sizes with a drastic reduction in time. Algorithm 3 gives the pseudocode for this approach.

**Algorithm 3:** Heuristic Horizontal Growth

*T_c_*[*i*, *a*] ← 0, ∀*i*, *a*
**while** some row is non-extended **do**
Find non-extended row *i* and value *a* so that $t_{n}=\left(\begin{array}{l} k-1\\ t-1 \end{array}\right)-T_{c}[i,a]$ is maximum
**if** *t_n_* = 0 **then**
stop horizontal growth
**end if**
Extend row *i* with value *a*
**for all** non-extended rows *j* **do**
*S* ← set of columns where row *i* and *j* have identical entries
$T_{c}[j,a]\leftarrow T_{c}[j,a]+\left(\begin{array}{c} \left|s\right|\\ t-1 \end{array}\right)$
**end for**
**end while**

### 4.2 Analysis

Notice that because we no longer search through any *t*-tuples, we no longer need the *Cov* array, so our space complexity is drastically reduced to just Θ(*rv*).

While the space savings are good, the particular point that makes this approach worthwhile is the speed gains. In particular, this sets $\bar{S}=0$ in the time analysis for the original algorithm. This allows a rigorous worst-case bound to be set forth of $O\left(r^{2}v+r\left(\begin{array}{l} k-1\\ t-1 \end{array}\right)\right)$ for one stage of horizontal growth. Combining this with the vertical growth stage over all iterations of the algorithm we get the bound $O\left(r^{2}vk+r\left(\begin{array}{l} k\\ t \end{array}\right)+rv^{t}\left(\begin{array}{l} k\\ t \end{array}\right)\right)$. Approximations for *r* are less valid here because for larger values of *v*, such as those larger than 10, the heuristic introduces too much error into the process. However, aside from those values, this method clearly demonstrates a fast heuristic that, as shown in Sec. 5.3, can also produce decently sized covering arrays.

## 5. Implementation and Empirical Results

### 5.1 Implementation

IPOG is currently implemented in a software package called FireEye [8], which was written in Java. The algorithms outlined in this paper were also implemented in Java as IPO′ and IPO″, with IPO″ the version with the heuristic horizontal growth algorithm. These implementations are currently incorporated into FireEye as IPOG-F and IPOG-F2, respectively. Both versions of horizontal growth use randomization to break ties in the greedy selection. While this seems undesirable because it lacks the repeatability of IPOG, which is deterministic, only minor differences in time and covering array size have been observed. However, this can still be important in a few notable cases. For example, with *t* = 5, *v* = 2, and *k* = 13, the smallest known size was previously 104 [2] but by running IPO′ many times and taking the minimum size, the new bound of 103 was generated. A more typical result would be around 112. This is not a drastic gain so, if needed, one could probably fix the seed for the pseudorandom number generator and still be very confident in behavior matching what is described in this paper.

As noted earlier, this paper will only talk about homogeneous alphabet covering arrays. The ideas scale to heterogeneous alphabets and the two implementations IPO′ and IPO″ can deal with these situations. However, the performance gains described in this paper do not seem to extend to this more general setting as while competitive results are seen, IPOG seems to do better.

### 5.2 Comparison to FireEye

In this section graphs are presented to compare FireEye running the IPOG algorithm and IPO′. All runs were performed on a 2.6GHz AMD Opteron machine with 4GB of RAM allocated to the programs. The following graphs compare FireEye and IPO′ only for the case *t* = 3 and *v* = 3, but these results are representative of the many runs observed for small values of *t* and *v*.

**Fig. 1:**
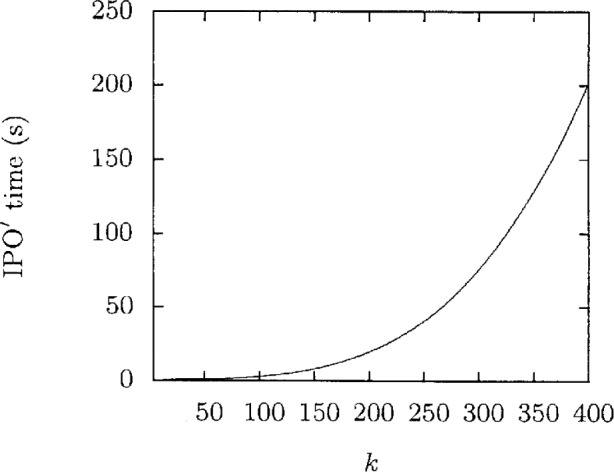
Execution time for *t* = 3, *v* = 3.

**Fig. 2:**
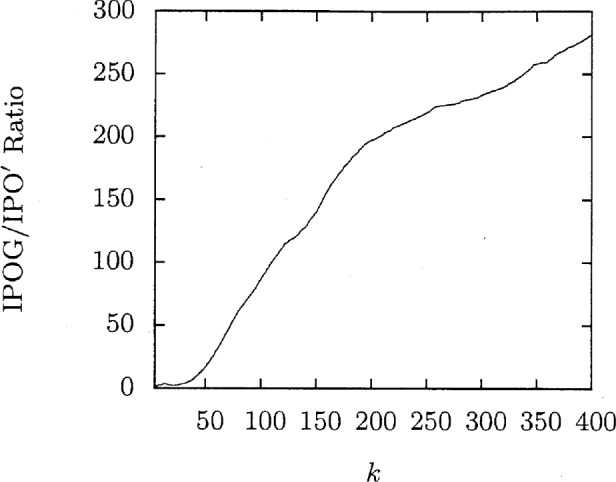
Time comparisons for *t* = 3, *v* = 3.

Figure 1 shows the amount of time IPO′ takes to generate each covering array. This time is rather modest. Figure 2 takes the IPO′ time as a normalizing factor for the time spent running FireEye for the same situation which gives a speedup ratio for IPO′. This ratio is greater than 1 except when *k* = 4 and greater than two for *k* ≥ 8. The ratio is significantly large for even modest values of *k* and is clearly increasing with *k*. The largest speedup ratio in this graph, 281, clearly shows the advantages of IPO′.

**Fig. 3:**
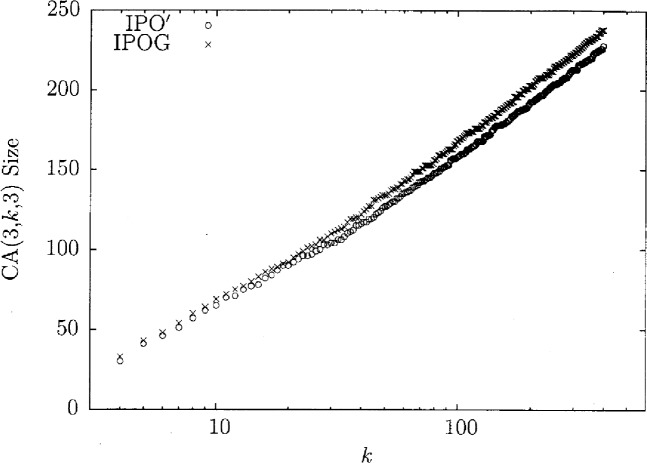
Size comparisons for *t* = 3, *v* = 3.

Figure 3 shows the sizes of the resulting covering arrays from IPO and IPO′. It is important to note that IPO′ seems to maintain around a 5 % smaller covering array when compared to FireEye. This 5 % is important because it allows IPO′ to produce the smallest covering arrays in the literature [2] for *k* ≥ 208. This fits with the intuition that by searching a larger space, IPO′ can achieve a more optimal result. These graphs show that IPO′ has both a time and optimality advantage for *t* = 3 and *v* = 3. Similar results have been seen in all situations observed thus far.

IPOG, and its implementation FireEye, have already shown to be competitive in both time and size comparisons with other algorithms so these results suggesting that IPO′ performs better than FireEye speaks well to its performance in general. We compare FireEye and IPO′ to the DDA, the Deterministic Density Algorithm [1]. For *t* = 2, *v* = 4 and *k* = 100, IPO′ gave a covering array of size 53 in 0.6 seconds. FireEye gave a covering array of size 54 in 2.3 seconds. DDA is reported to give an array of size 51 in 24.9 seconds. While IPO′ may not give the best array size, the time savings are significant.

### 5.3 Heuristic Performance

**Fig. 4:**
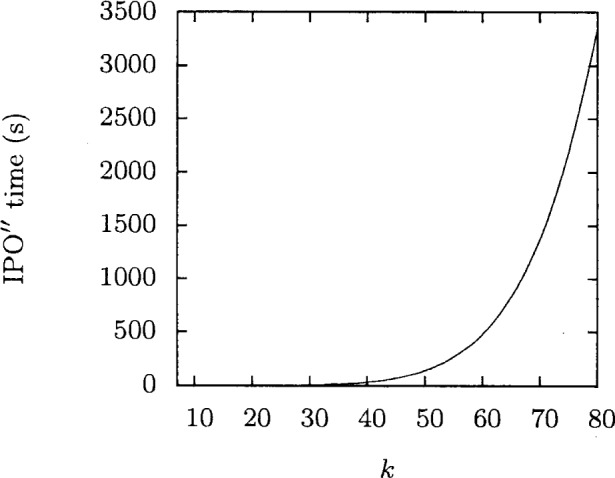
Execution time for *t* = 6, *v* = 2.

**Fig. 5:**
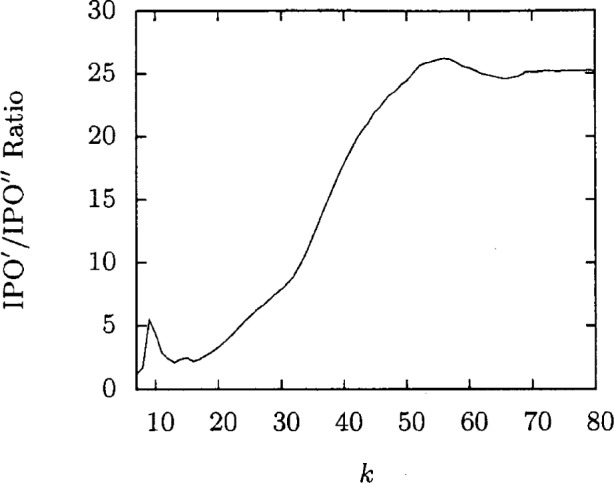
Time comparisons for *t* = 6, *v* = 2.

**Fig. 6:**
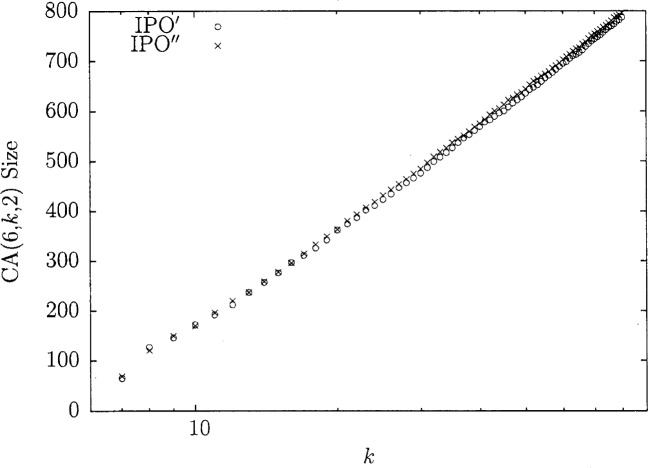
Size comparisons for *t* = 6, *v* = 2.

It has been shown that IPO′ is very efficient, but for extremely large covering arrays such as when *t* = 6, it still requires more time than might be feasible. The heuristic from above was theoretically shown to be much faster than the original idea. By implementing the heuristic as IPO″, we have observed that it is competitive in size for small values of *v*. We demonstrate that fact in the case that *t* = 6 and *v* = 2. Figure 4 shows the amount of time IPO″ takes as a function of *k*. In Fig. 5 we show the time IPO′ took normalized by execution time IPO″. With these two graphs we see that the heuristic offers major time savings. While the time savings are important, it is also key that this gain does not drastically increase the size of the resulting covering array. In Fig. 6, the array sizes are shown. This graph shows how in this case the covering array resulting from the heuristic is not significantly larger than the array produced by the original idea. This suggests that the guess of taking $T_{c}[j,a]\leftarrow T_{c}[j,a]+\left(\begin{array}{c} s\\ t-1 \end{array}\right)$ is a good one and not too far from the actual value. It is worth noting that for larger values of *v*, the error introduced by our guess was too much to be practical and can be large enough to make IPO″ run slower than IPO′. It should be noted that for 38 ≤ *k* ≤ 80 the covering arrays generated by IPO′ are the smallest known, as compared to [2].

### 5.4 Covering Array Numbers

**Fig. 7:**
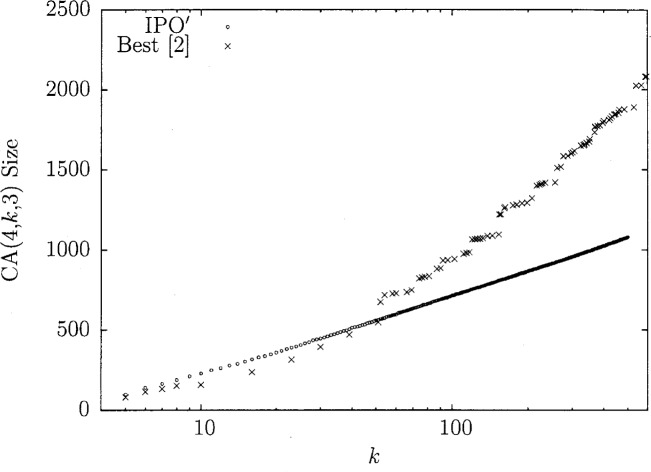
Covering array numbers for *t* = 4, *v* = 3.

IPO′ has been run for many small values of *v* and *t* for as large *k* as possible. In many situations, for large *k*, IPO′ has created the smallest known covering array sizes, some of which have been noted already. In Fig. 7, the results for IPO′ are compared with the best known numbers [2] in the case that *t* = 4 and *v* = 3. Notice how for values 52 ≤ *k* ≤ 500 (and for some *k* < 52), IPO′ gives the best known covering array size seen in the literature and further how the covering array size seems fairly linear in this log-plot, suggesting this algorithm does very well asymptotically. This entire data-set took three weeks to generate but reached *k* = 52 in 31 seconds. All of these covering arrays were saved and are available upon request.
